# Wave2Vec: Vectorizing Electroencephalography Bio-Signal for Prediction of Brain Disease

**DOI:** 10.3390/ijerph15081750

**Published:** 2018-08-15

**Authors:** Seonho Kim, Jungjoon Kim, Hong-Woo Chun

**Affiliations:** 1 Convergence Research Center for Diagnosis, Treatment and Care System of Dementia, Korea Institute of Science and Technology (KIST), 02792 Seoul, Korea; seonhokim@gmail.com (S.K.); jungjoonkim@kisti.re.kr (J.K.); 2 Korea Institute of Science and Technology Information (KISTI), 02456 Seoul, Korea; 3 Science and Technology Information Science, University of Science & Technology (UST), 34113 Daejeon, Korea

**Keywords:** electroencephalography (EEG), bio-signal, prediction of brain disease, Wave2vec, alcoholism, dementia, sequence classification, deep learning

## Abstract

Interest in research involving health-medical information analysis based on artificial intelligence, especially for deep learning techniques, has recently been increasing. Most of the research in this field has been focused on searching for new knowledge for predicting and diagnosing disease by revealing the relation between disease and various information features of data. These features are extracted by analyzing various clinical pathology data, such as EHR (electronic health records), and academic literature using the techniques of data analysis, natural language processing, etc. However, still needed are more research and interest in applying the latest advanced artificial intelligence-based data analysis technique to bio-signal data, which are continuous physiological records, such as EEG (electroencephalography) and ECG (electrocardiogram). Unlike the other types of data, applying deep learning to bio-signal data, which is in the form of time series of real numbers, has many issues that need to be resolved in preprocessing, learning, and analysis. Such issues include leaving feature selection, learning parts that are black boxes, difficulties in recognizing and identifying effective features, high computational complexities, etc. In this paper, to solve these issues, we provide an encoding-based Wave2vec time series classifier model, which combines signal-processing and deep learning-based natural language processing techniques. To demonstrate its advantages, we provide the results of three experiments conducted with EEG data of the University of California Irvine, which are a real-world benchmark bio-signal dataset. After converting the bio-signals (in the form of waves), which are a real number time series, into a sequence of symbols or a sequence of wavelet patterns that are converted into symbols, through encoding, the proposed model vectorizes the symbols by learning the sequence using deep learning-based natural language processing. The models of each class can be constructed through learning from the vectorized wavelet patterns and training data. The implemented models can be used for prediction and diagnosis of diseases by classifying the new data. The proposed method enhanced data readability and intuition of feature selection and learning processes by converting the time series of real number data into sequences of symbols. In addition, it facilitates intuitive and easy recognition, and identification of influential patterns. Furthermore, real-time large-capacity data analysis is facilitated, which is essential in the development of real-time analysis diagnosis systems, by drastically reducing the complexity of calculation without deterioration of analysis performance by data simplification through the encoding process.

## 1. Introduction

Studies are being actively carried out for diagnosis, classification, and even prediction of diseases by using computer technologies and the analysis of vast accumulated data in the bio-health field. One of the major research areas in the data analysis-based disease prediction field is prediction and analysis of diseases, which focuses on discovery of major features in the medical data. Using such data, analysis is carried out to reveal the effects of respective features on a certain disease. For example, Reference [1] studied vitamin C inadequacy in diabetics, associated with glycemic control, obesity, and smoking. Reference [2] tried to find the effective duration of past disease referencing in predicting dementia using the national health insurance database. In addition to these data analysis research trends in the bio-medical field, many researchers are concentrating on applying deep learning—the most advanced of the artificial intelligence techniques—effectively for prediction and diagnosis of disease.

Electroencephalography (EEG), the electrical bio-signal obtained from brain, has been a source of important information to many researchers to predict and diagnose diseases and status of the brain for a long time. EEG research mainly includes (1) a method of separating the frequencies that compose the EEG through Fourier analysis [3], thereby measuring the content of each frequency region; and (2) a method of analyzing the waveform of EEG for a certain duration of time. Furthermore, depending on the measurement technique, there are methods of measuring the EEG resting state or spontaneous potential (SP), and a method of measuring the EEG induced by the stimulation of vision, hearing, and touching or evoked potential (EP) [4,5]. Furthermore, there are methods of conducting research for the types and locations of EEG occurring according to the disease and stimulation, and the relationship and connectivity between brain regions of EEG occurrence. Recently, studies on the application of deep learning techniques have been carried out for diagnoses of brain diseases, such as schizophrenia, seizures, depression, insomnia, dementia, and alcohol addiction, as well as studies for brain–computer interactions [6,7] through unsupervised feature extraction from EEG [8,9]. However, the analysis conducted through deep learning for bio-signal data, such as EEG and electrocardiogram (ECG), is basically a study for extraction of unsupervised features by inputting real number time series data based on the time axis in the neural network. Not only is the complexity of calculation large for a large amount of real number processing, but also the interpretation and understanding of the analysis process or results are not easy. Furthermore, searching, representing, and identifying effective patterns are not easy. These problems are common in deep learning [10], but they are issues that must be resolved for applications in the medical field, in which the grounds for major decision-making and explanations for reasoning processes are important.

To resolve these issues for processing of real number time series data, such as bio-signals in deep learning, the motivations of this study are summarized as follows: (1) to compose the feature extraction process of the machine learning stage more intuitively and facilitate easy explanation and understanding of the analysis process; (2) to facilitate recognition and identification of effective patterns for analysis; and (3) to facilitate the foundation for a high-speed large-capacity analysis system by reducing the complexity of calculations through simplification of data without sacrifice of analysis performance.

Vectorization of information is essential for performing comparisons or facilitating basic computations of data, such as gene data, clinical data, and document data. Vectorization is a necessary method of representing information for status display and basic computation (comparison). For example, the data used for prediction of diseases include temporal information expressed with classification values or explicit numbers, such as family history, clinical data, and demographic data; but they also include bio-signal data, which are temporal continuous numerical data, such as EEG and ECG. In most of conventional classification studies [11,12], feature vectors, which were formed by extracting and combining temporal information from data, were used for comparison and computation of data, but a lot of research is still needed for the vectorization method of real number time series bio-signal data.

A problem like prediction and diagnosis of diseases through analysis of bio-signals can be viewed as a sequence classification [13] problem, which is one of major problems of data mining [14]. Sequence classification shows problems with conducting categorization by using continuous data of a monotonous simple type. Furthermore, there are problems where feature dimension is high and extraction is difficult because of the lack of an explicit feature, unlike usual classification tasks, for which experts compose and use feature vectors for classification. Therefore, sequence classification is a good application field that can be used in prediction and diagnosis of diseases by directly learning the bio-signal data, while eliminating the expert-intervened feature vector extraction stage through application of deep learning.

There are conventional studies that have been conducted for seizure prediction by extracting the feature vectors, such as statistical features, fractal features, and entropy through signal analysis from EEG [15], but not many studies have tried to vectorize the time series data. Recently, Ref. [16] vectorized the signal data itself and applied it to deep learning. They proposed a method of learning left and right context information by using the neural network (continuous bag of words: CBOW) [17] after obtaining final embedding by combining the demographic information with the inherent latent, which were extracted with an unsupervised method using sparse autoencoder (SAE) for EEG of fixed length. However, like the conventional deep learning systems, such a system also has the problem of difficulty explaining and understanding the process and results of analysis, because major procedures—such as feature extraction that uses SAE and pooling, and learning that uses weight modification of network nodes—remain black boxes.

Unlike the connectionist approach, such as neural networks or deep learning, the statistical symbolic approach is a method that deals with correlations with emerging patterns for symbols that are forming the sequences, and it is much more convenient to understand [18,19]. Typical examples include genomic sequence analysis, music search by scale, or natural language processing using computers. If the real number time series data, such as EEG and ECG, are represented as a sequence of symbols like sentences and learned and used for analysis, they would be helpful when explaining and understanding the causes or results of feature selection, training, pattern recognition, and classification. Furthermore, if unnecessary information is excluded and only the necessary information is left in the symbolization stage, the complexity of computation will decrease, and in some cases, improvement of analysis performance can be expected.

In this study, an encoding-based Wave2vec time series classifier model is proposed, which applies deep learning whereby the data are vectorized and the black boxes of the deep learning stage are minimized by converting the classification problem of real number time series data, such as bio-signals, into a sequence classification problem of symbolic approach.

In Section 2, the proposed model is described in more detail; in Section 3, explanations are provided for the experimental results with respect to the usefulness of the model, optimal parameter search, and performance comparison with the conventional deep learning systems. Finally, in Section 4, discussion and conclusions are provided.

## 2. Encoding-Based Wave2Vec Time Series Classifier

The Wave2vec model proposed in this study vectorizes the bio-signals of a time series type. It is like the Word2vec, which is a natural language modeling technique that uses deep learning, in that it performs computations for words by vectorizing the words of natural language. The modeling of signal classes is facilitated using deep learning through vectorization of bio-signals, and these models are used for prediction and diagnosis of diseases, as well as extraction of effective patterns inside the bio-signals. To vectorize the bio-signals, after changing the signals to a sequence of symbols, such as gene information or natural language by using encoding techniques, the emerging patterns of signal patterns in the sequence are learned using deep learning, thereby modeling the bio-signals for each class. Such an encoding-based Wave2vec model implements the concept of signal classification via probability-based pattern recognition with the deep learning technique. In this section, detailed descriptions are provided for the theoretical components of the methodology as follows, with which the Wave2vec model performs diagnosis of EEG: (1) signal encoding, (2) sequence classification, (3) wave embedding and wave vector, and (4) diagnosis and prediction through vector computation.

### 2.1. Signal Encoding

To apply a symbolic-data-based deep learning approach like Word2vec for continuous real number time series data, such as bio-signals, an encoding process is required, which converts the signal data to a sequence like text string. Encoding was originally used in data communication, compression areas, or sound processing areas. It digitalizes the analogue data of wave-type bio-signals. However, in this study, it was applied to convert wave-type digital bio-signals to a sequence of symbols. If the bio-signals of text string type converted into a sequence of symbols are vectorized using deep learning, comparison and computation among bio-signals become possible, and they can be used for model creation and diagnosis.

**Figure 1:**
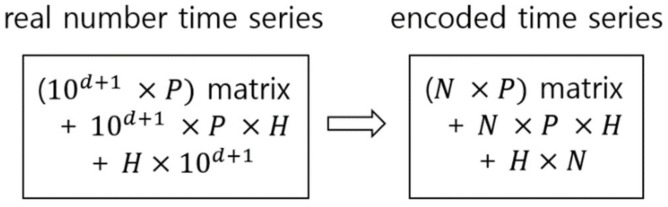
Complexity reduction in pattern learning through encoding the real number data. *N* is the base number of encoding (*baseN*), which is the size of a targeted symbol set; *d* is the number of digits of concern after the decimal point; *P* is the size of projection layer; and *H* is the size of hidden layer.

Processing encoded signal data through a symbolic approach has some advantages over directly processing the real number time series data. (1) It is easy to explain and understand the analysis process and result by increasing readability. Data represented as a symbol list, like gene sequencing, is easier to read and understand than the real number time series. (2) Pattern recognition and search within the signals are easy. Recognition and search are difficult for the emerging pattern information of real numbers. (3) Encoding reduces the complexity of calculation and increases the analysis speed by simplifying the data. It is very difficult to extract the emerging patterns of data from the real number time series, and if they are extracted by treating each real number as a single instance, the complexity of calculation will increase exponentially according to the increased number of decimal places expressing the real numbers. Figure 1 shows an example of how the complexity of calculation decreases by encoding the real number time series data to an encoded time series, which is a sequence of symbols, when performing pattern learning when the emerging patterns of real numbers are learned using deep learning. In this calculation, using a conventional deep neural network and learning emerging patterns for recognition and identification of effective patterns as a common task are assumed for both data types.

Reduction in calculation complexity is a problem that must be solved in order to obtain fast response time of a real-time analysis system and analysis of large-capacity data, such as sleep EEG.

Furthermore, encoding can improve the analysis performance by excluding unnecessary detailed information. Encoding inevitably leads to some data loss, but it does not always cause deterioration of analysis performance. In fact, in the areas of voice recognition, face recognition, etc., a data refinement stage is included, which simplifies most data and performs noise removal, among others, to enhance the accuracy of analysis. For example, in the study of [20], voice recognition rate was increased through reduced bandwidth analysis, and in the studies of [21,22], down-sampling was included in the preprocessing process to improve the efficiency and accuracy of feature extraction.

In natural language, word types are limited to the number of words in the vocabulary dictionary. On the other hand, for EEG data, because the EEG data are a series of real numbers based on a time axis, there are infinitely many types and, thus, if learning is conducted right away, it will not proceed well because the calculation complexity is high; even if it does, there may be a problem of overfitting, whereby the entire data are learned without allowing flexibility. To solve this problem, it is necessary to perform transformation mapping from EEG fragments of fixed length to one of the pattern sets of fixed numbers through encoding.

The encoding method used in our proposed Wave2vec model is combined with a method of quantizing a bio-signal fragment of fixed length—a wavelet whose length is defined by the sampling rate—to one of the symbols of a fixed number, namely, bag-of-symbols, through simple computation. As the number of symbols allowed in the encoding is decreased, the data becomes more simplified and the calculation complexity is decreased, but data loss will become severe, thus analysis performance will deteriorate. On the other hand, if the number of allowed symbols is increased, the data will become similar to the original bio-signals, and the effect of encoding will disappear. Therefore, it is important to find a tradeoff between the calculation complexity and analysis performance.

**Figure 2:**
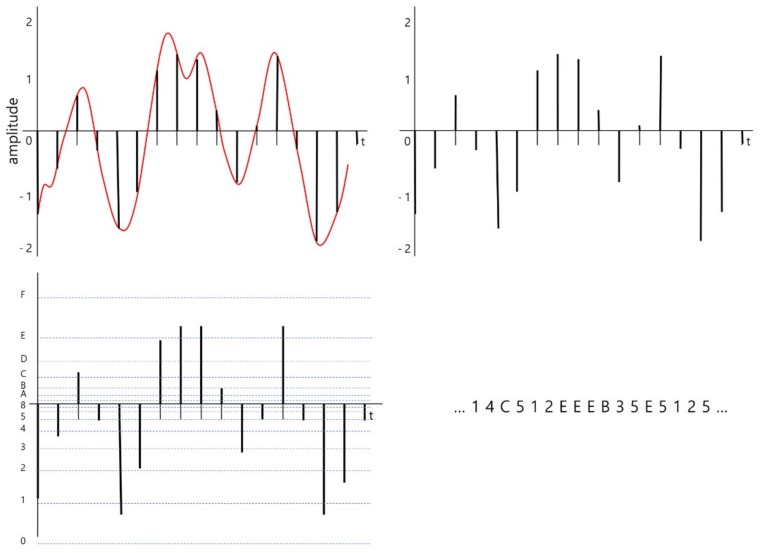
Process of converting an electroencephalography (EEG) bio-signal into a sequence of symbols. Sampling the original signal (**top left**), sampled signal (**top right**), quantized signal (**bottom left**), and symbolized signal (**bottom right**). *baseN* = 16.

The brief encoding processes are illustrated in Figure 2. The original continuous signal is converted into a discrete signal with an identical time interval during the sampling stage. The time distance between sampled signals is defined by a given sampling rate. In our system, sampling rate is decided by the user. Then, the amplitudes of the sampled signal are quantized into near symbols. In this figure, 16 is used as a base number, *baseN*, of the encoding, and the 16 target symbols are hexadecimal letters from ‘0’ to ‘F’. The result of encoding the signal is a sequence of symbols.

When encoding bio-signals, the variables to consider include sampling rate, number of amplitude levels, and input voltage range, which must be properly adjusted. There are various encoding techniques, such as performing quantization by dividing the input voltage range (range of values that normalize and correct the sampling rate data of fixed time duration) into the sections of a fixed number (number of amplitude levels), or a method of assigning symbols to data values through arbitrary simple computation [23]. Although we could not test all advantages/disadvantages of various encoding techniques in the EEG analysis area, in this study, we decided to perform the encoding through quantization after extracting the EEG change value information with the delta encoding method [24]. This was because the deviation is large for the distribution of amplitude of EEG, whereas the deviation is small for the amplitude change values of EEG, thereby facilitating the encoding of smaller dimensions. Other reasons for using delta encoding for EEG change values as a feature are: (1) because delta encoding stores only the amplitude change values of the original data, the change range is not large and, thus, the number of target words can be drastically reduced; (2) by reducing and smoothing the value range, the overfitting possibility can be reduced for abnormal noise signals in the deep learning stage; (3) as only the differences between adjacent data are recorded, changes during a certain time period can be stored, instead of storing temporal information of a certain moment; and (4) difference of absolute measurement values according to uneven sensitivity between sensors can be reduced, and hardware jittering can be reduced.

**Figure 3:**
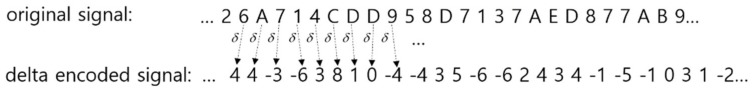
Concept of delta encoding. Amplitude changes between sampling intervals are the result of encoding.

The concept diagram of delta encoding is shown in Figure 3.

In other words, only the amplitude change information of a certain time duration (one tick in the example of Figure 3) is extracted from the raw data value (amplitude of EEG) information, and the encoding is carried out through quantization by dividing the output range into the number of delta levels. The bottom left section of Figure 2 shows how quantization is performed.

**Figure 4:**
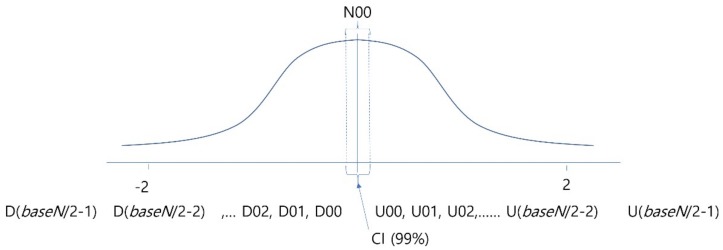
Analyzing overall data distribution for deciding quantization interval and labeling.

For deciding on the degree of quantization, it is necessary to identify the overall distribution of EEG amplitude changes beforehand, which is shown by the Gaussian curve in Figure 4. The symbols, from *D*(*baseN*/2–1) to *U*(*baseN*/2–1), below the curve represent the symbols to be mapped to the values of the corresponding section. In other words, to encode the data of a given time section, the normalized EEG change values of the corresponding section were divided into the stages of *baseN*, which is an encoding base number, based on the Gaussian distribution, and converted to *baseN* unique symbols. To help the readability of symbols, symbols beginning with ‘*U*’ were used for the case of the amplitude value increasing, while the symbols beginning with ‘*D*’ were used for the case of decreasing values. The data in sections smaller than standard deviation of −2.0 or greater than 2.0 were regarded as noise or results of machine malfunction, were no longer divided into specific stages, and were corrected using a method of combining them in one stage. This is because if learning is performed without correction of these parts, overfitting may occur and good performance will be shown only for the training data, and the analysis performance will decrease for data other than the training data. Furthermore, because the normalized EEG’s change values are very small, ‘*U*00’ and ‘*D*00’ sections, i.e., the center parts corresponding to the statistically confident interval, are not divided into two sections of plus and minus, and are combined into a single section so that the noise occurring due to slight jittering of sensors will not be learned.

### 2.2. Sequence Classification

The problem of predicting the onset of brain disease from EEG data represented as a sequence of symbols is a kind of sequence classification [13], and it is the same as sentence classification in statistical artificial intelligence. Studies, especially those involving sentiment analysis, are actively carried out for the classification of given sentences according to their meanings by learning the emerging frequency, co-occurrence, emerging order, and context of words emerging in sentences.

Document classification, another instance of the sequence classification problem, is performed by using the order of words as a major feature [25], a sentimental analysis is performed by using the sentiment lexicons of words [26], and the pattern-based sequence classification is studied in more general areas [27]. In the study by [28], the class classification probability of sentences, which is a kind of sequence, was expressed with the following equations using the class probability of patterns composing a sentence:

(1) $$class\;of\;a\;sequence\;=arg\underset{c}{\max}p\left(c|sequence\right),$$

(2) $$p\left(c|sequence\right)=\frac{\sum_{pattern\;}^{}\left(O\cdot S\cdot p(c|pattern)\right)}{n},$$

(3) $$p\left(c|pattern\right)=\frac{L}{G\;},$$

where a pattern is a set of words in the sentence of arbitrary length. *O* is the number of occurrences of the pattern in the sentence, and *S* is the pattern weight proportional to the *N* value of *N*-gram—the length of the pattern—which is included to give some privilege to longer patterns. *n* is the number of words in the sentence, *c* is a class, *L* is the frequency of the pattern used as class *c* in the whole training data, and *G* is the frequency of the pattern, whose length is *N*.

In other words, a sentence consists of patterns, which are combinations of words, and if prior knowledge has been built for the emerging probability by class for each pattern, the class of a given sentence can be determined by analyzing which patterns are composing the sentence.

We assumed that such a probability-based classification theory of text learning, which is a statistical symbolic approach, can be directly applied to all types of time series data, such as the bio-signals of EEG and EEC, and even financial data, climate changes, and trend changes of social network service (SNS). However, in this paper, we focused only on the learning of bio-signals. That is, by dividing the bio-signals into patterns, which are combinations of wavelets that compose the bio-signals, and by learning the probability and type of each emerging pattern for each class, they can be used in determining the classes for arbitrary bio-signals.

In our study, nevertheless, unlike the case of text learning, learning was conducted by using the deep learning technique, instead of investigating or calculating basic information or prior probability one by one for emerging probabilities for a respective class of pattern. Through learning, a wave vector is obtained for each pattern, and a model is created for each class through vector calculation, in which the wave vector is applied to each class data. The model of each class calculates the suitability for arbitrary new signals to itself, and the classification is performed through comparison for the suitability of other models. In the next subsection, the methods of vector operations employed in our model for developing wave vectors, learning EEG classes, and classifying arbitrary data are described.

### 2.3. Wave Embedding and Wave Vector

The methods of representing certain data include (1) sparse representation (one-hot representation), which represents all attributes that can be possessed by the data, with respective independent dimensions, and represents it as one if there is an attribute and zero if not; and (2) dense representation (i.e., embedding), which represents data by corresponding the dimension of a fixed number to the data. With the sparse representation, comparison or calculation of data is impossible, but comparison and basic operation of data are facilitated via embedding. If the words “hotel” and “motel” are represented with sparse representation, as there is no common attribute between the two words, they are words unrelated to each other. However, if “hotel” and “motel” are represented by embedding with n dimensional vectors, the similarity of two words can be numerically represented and, therefore, it can be computed that the relationship of “hotel” and “motel” is closer than the relationship of “hotel” and “pineapple.” Such an embedding technique applied to word representation of natural language is Word2vec, and that applied to representation of time series data of a wave type, such as EEG, EEC, financial data, and climate data, is Wave2vec.

Wave2vec facilitates not only the vector representation of patterns, which are made by wavelets and wave fragments of a fixed time length in bio-signals, but also the vector representation of entire bio-signals. Moreover, the comparison, clustering, classification, and basic operation of bio-signals are facilitated. A traditional sequence representation model, *tf-idf* model [29], also has a commonality whereby comparison computation is facilitated for the patterns, but unlike *tf-idf*, which represents the importance of a pattern as a number by statistically analyzing the emerging frequency in data, Wave2vec represents the importance as a vector by learning the co-occurrence between patterns; thus, it can be said that its usability is higher and suitable for representing patterns in bio-signals.

In this study, to learn the sequence of symbolized wavelet emerging patterns, the continuous bag-of-words (CBOW) model [17] was used, which is a neural net architecture that predicts a center wavelet by referencing the adjacent context wavelets. The CBOW algorithm is capable of learning the contexts of words and is commonly applied to text classifiers, as [30] used it for classifying healthcare tweets.

**Figure 5:**
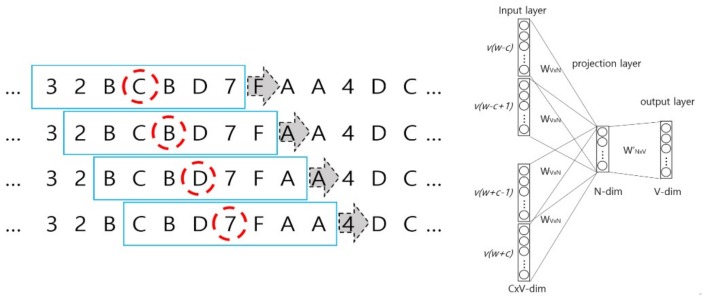
Shifting windows on the sequence of symbolized EEG signal for continuous bag of words (CBOW) feeding (**left**) and CBOW architecture (**right**).

The left panel of Figure 5 illustrates the CBOW algorithm in the Wave2vec that performs the embedding of patterns (fragments of signal), while the right panel of Figure 5 shows the architecture of CBOW. In the example shown in the left figure, a single signal is composed of *C* wavelets, each fragment is encoded with hexadecimals, and the length of sliding window *t* = 7 on the encoded symbol sequence. In the windows represented as blue rectangles, the center of a window is the target symbol marked by a red dashed circle, and learning is repeated to predict the target symbol from a total of six context symbols, i.e., three symbols each, (*t* − 1)/2, on the left and right sides. From one window, *t* input data are produced for the CBOW inputs, and as the window slides to the right one symbol at a time, CBOW inputs are filled continuously. As learning is conducted in this manner for the entire symbol sequence, the weights on the deep neural network are continuously revised, and finally, the set of weights that produces the minimum error is determined. In the inputs of the CBOW architecture diagram, *t* words for each of *C* windows are inputted as one-hot encoded representation, and by using a *V* × *N* projection matrix, the average of each input vector is sent to the projection layer where, by multiplying *N* × *V* weight matrix, it is sent to the output layer. Here, *V* and *N* represent the total number of vocabularies and number of dimensions of the desired target vector in the system, respectively.

**Figure 6:**
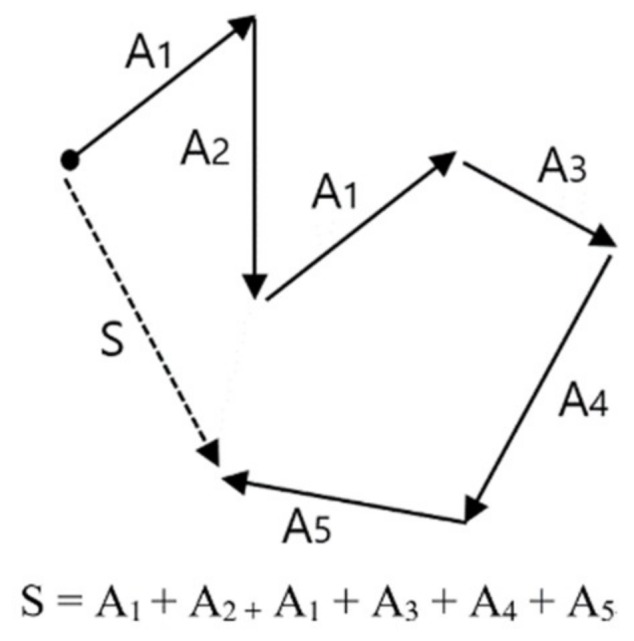
Concept of vector summation, which is the basic operation of vectorized EEG learning.

The result of wave embedding is a wave vector table, which is a set of wave vectors for all wavelets or patterns existing in all signal data. Once the wave vector table for EEG is produced, we can make the desired trained model by learning the training data we want. The training data refers to the labeled data of a certain amount or more. The learning of training data with a vector table is carried out with a method of calculating vector summation or vector average of wave vectors in all the signals composing the training data. Figure 6 explains the basic concept of vector summation. In this example, vector S—which is the vector summation of all training data A1, A2, A1, A3, A4, A5—is the learning result of these training data. In the result of learning using vector summation, only the direction of vector S is significant, and as the magnitude of vector is not considered, it does not matter whether the vector summation or vector average is used for the learning method.

Previous approaches of time series data classifiers based on deep learning normally consist of an encoder, such as a sparse autoencoder that works as an automatic feature extractor; multiple deep learning layers, such as a convolutional neural network (CNN), recurrent neural network (RNN), and deep neural network (DNN); and a softmax at the last stage that outputs the probability of classification to each possible class. Our proposed encoding based on the Wave2vec time series classifier also includes deep learning layers. However, unlike other systems, our system employs a tokenizer that accepts some parameters to conduct encoding by the selected feature and parameters variant to the data type, sampling for variable wavelet length, and normalization. That is, the tokenizer is a module that customizes the classifier to its applications, such as EEG and ECG analysis, finance prediction, and weather forecasting, by selecting the encoding feature and parameters. Moreover, the Wave2vec time series classifier does not output classification or include a softmax because it does not classify the testing data directly. Instead, it produces as many models as the number of output classes. Actual classification is done by separate offline procedures.

### 2.4. Vector Operation for Prediction and Diagnosis

After finishing the learning, if the model by class, i.e., the dementia EEG class model and the normal EEG class model, is made, prediction of onset or diagnosis can be carried out for an arbitrary testing EEG by using these models. With such a method of creating class models, if the patterns composing the testing EEG, i.e., wavelets, are learned, the wave vector for the testing EEG can be obtained, and by comparing this vector with the model of each class, the similarity between the EEG and the model of each class can be calculated. Thus, the prediction or diagnosis is carried out with the class that is finally determined as the closest model.

Here, the comparison between vectors can be conducted by using the angular distance between two vectors, and it can be calculated with cosine similarity. As shown in the following equation, the similarity between two vectors *A* and *B* is deduced from the calculation of the dot product of *A* and *B*:

(4) $$similarity=\cos\left(\theta \right)=\;\frac{A\cdot B}{||A||||B||}=\frac{\sum_{i=1}^{n}(A_{i}\times B_{i})}{\sqrt{\sum_{i=1}^{n}{\left(A_{i}\right)}^{2}}\times \sqrt{\sum_{i=1}^{n}{\left(B_{i}\right)}^{2}}},$$

where *A* and *B* are vectors, and *A_i_* and *B_i_* are the *i*-th components of *A* and *B*, respectively.

According to this equation, the cosine similarity becomes close to one as the directional angle of two vectors on the *n* dimensional vector space becomes similar; close to negative one as it becomes close to the opposite direction; and close to zero as it becomes close to perpendicular.

**Figure 7:**
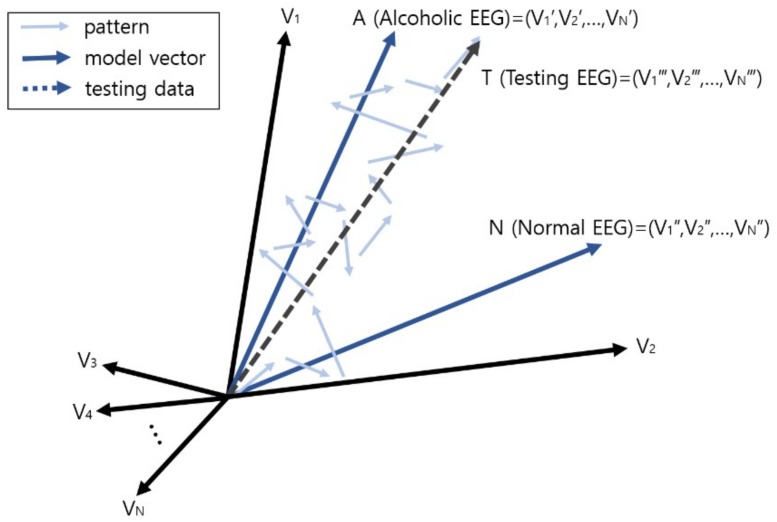
Prediction or diagnosis of brain disease through vector comparison.

Figure 7 depicts the process of classification, i.e., diagnosis, of an alcoholic EEG through vector comparison, and shows the vector comparison computation between the vector sum T for each pattern in the testing EEG and the two learning model vectors A and N. In this example, as the wave vector T of the testing EEG is closer to the alcohol EEG model A, this EEG is diagnosed or predicted as alcohol addiction.

In the next section, a series of experiments and results are presented to prove the advantage and usefulness of the encoding-based Wave2vec time series classifier. An experiment for investigating the optimal encoding parameters by testing the changes of brain disease prediction accuracy according to the number of encoding target symbols, i.e., encoding resolution, for the EEG signals of humans is described. Subsequently, the results of the performance comparison experiment are discussed for the proposed model and the conventional deep learning classifier with network-based deep learning algorithms, such as CNN, RNN, and DNN. Furthermore, an experiment of model visualization to recognize effective patterns for diagnosis and to identify the actual patterns is also described.

## 3. Experiments and Results

In the proposed Wave2vec model, deep learning is performed after converting the continuous real number data of a wave type to sequences of a series of simple symbols through data encoding. Therefore, while there is a decrease in calculation complexity and readability improvement of the analysis stage and results, as data loss is inevitable due to data simplification, we decided to confirm its effect on the analysis performance through the experiments. The data loss does not always lead to a negative result with respect to data analysis or pattern recognition. It not only reduces the size of data in the areas of voice recognition, image recognition, and EEG analysis, but it also allows the learning system to focus on major information only. Moreover, performance improvement is sought through down-sampled data or decreasing the range of analysis to prevent over-training for unnecessary specific information, noises, jittering, etc. In this section, three experiments are performed, and the results are provided for validation of the proposed encoding-based Wave2vec time series classifier by searching for optimal encoding parameters, comparing their performance to those of classifiers that use the conventional deep learning models, and demonstrating the recognition and identification of readable important patterns through the visualization of class models that are the result of the proposed model.

### 3.1. Data Description

**Figure 8:**
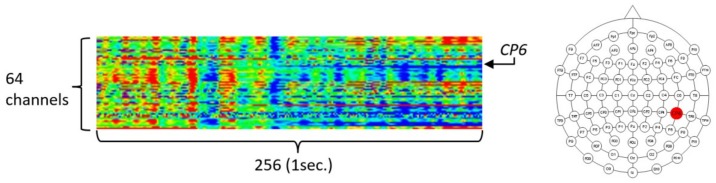
The structure of an instance of University of California Irvine (UCI) EEG data (**left**) and location of the CP6 electrode (**right**).

To validate the usefulness of the encoding-based Wave2vec time series classifier proposed in this paper for the biomedical area, three experiments were carried out by using the University of California Irvine (UCI) data mining repository’s EEG dataset [31,32], which is a real-world benchmark bio-signal dataset. The UCI EEG dataset is EEG data measured from a total of 122 subjects, and among them, 77 are alcoholic patients and 45 belong to the normal control group. These data are transient evoked potential, which are recorded EEG responses for the duration of 1 s, after stimulation by one of the three types of visual stimulation. By regarding the data as acquired in one instance, which was obtained by measuring the EEG triggered by a single stimulation, 120 measurements were taken per person, and thus, there were 120 instances, but because 120 instances were not reached for some experiment subjects, the data consisted of 11,057 instances. The total size of UCI data in the file system is 1,588,259,248 bytes. One EEG instance has 64 channels and a 256 Hz sampling rate, is of a 64 real number time series type recorded for 1 s, and is represented as a 256 × 64 real number matrix. The left panel of Figure 8 shows the structure of an instance of the data, which is in the form of a 256 × 64 matrix, through heat map visualization. The 64 data channels consist of 61 channels of EEG data, 2 channels of electrooculography (EOG) signals, and 1 channel of a reference signal. To provide insights into the UCI EEG data, heat map visualization was used by assigning a color spectrum from blue, for the lowest amplitude, to red, for the highest amplitude, after normalization within the −1.0 to 1.0 range.

In this research, we conducted experiments to compare the analysis performance, analysis speed, and effective pattern extraction capability of our Wave2vec time series classifier for the time series data with those of the conventional deep learning algorithms. For the experiments, we decided to select and use only one influential channel rather than using all 64 data channels, because such an analysis procedure—using only effective channels—is a commonly used research procedure for EEG analysis and we need to match our experimental results with those of previous research for comparison. In the experiment of [33], where the same data were studied, it was mentioned that the EEG of the CP6 electrode was the most effective for classification accuracy compared with the other selected 13 electrodes. Furthermore, it provides the accuracy measures of CP6 when conventional data mining techniques, such as *k*-nearest neighbors (*k*NN) and support vector machine (SVM), are applied. Referencing this, we selected the CP6 channel only, which was the 20th channel, in our experiments. The position of the CP6 electrode in the International 10/20 Electrode System is the same as that in the right-side image highlighted in Figure 8. The size of the CP6 channel data in the file system is 44,672,000 bytes.

### 3.2. Experimental Setting

**Figure 9:**
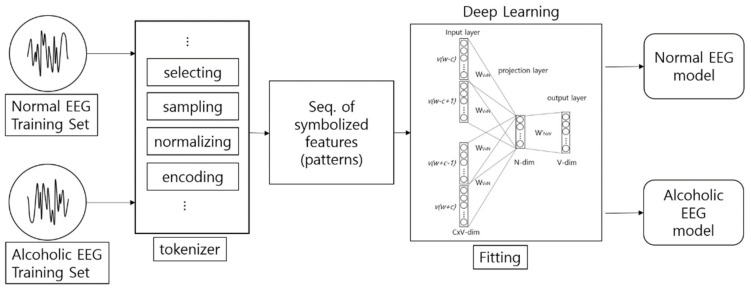
Architecture of the encoding-based Wave2vec time series classifier.

Figure 9 shows the architecture of the encoding-based Wave2vec time series classifier, which is proposed in this paper. This system has a structure of creating EEG models by classes from training data, i.e., normal class and alcohol addiction class. The classification for new data for testing is conducted offline of this system, and is carried out by calculating the fitness between the input data and each model.

The three EEG classifiers implemented with deep learning, which will be compared with proposed model, were implemented by using CNN, LSTM (Long Short-Term Memory Units, a variation of RNN), and DNN algorithms, which are widely accepted in general. The architectures of these systems are shown in Table 1. The ‘transpose’ modules rearrange the data so that they can be fed into the learner in temporal order, and the ‘dropout’ module deteriorates the efficiency of learning to reduce overfitting in the neural network.

The proposed encoding-based Wave2vec classifier also includes a deep learning layer (CBOW) like these three deep learning models, but unlike these models, which accept the real number data as they are and perform the auto feature selection using the sparse autoencoder (SAE), it actively selects and limits the range of learning features through encoding, and the deep learning layer only learns the sequential order of symbol patterns and their context, which is a simultaneous emerging relationship. The three deep learning EEG classifiers were all implemented in *Keras* [34], which is a deep learning library that uses TensorFlow as the back-end; the proposed Wave2vec time series classifier was implemented with DeepLearning4J [35], which is java-based open-source, distributed, deep learning library. The hardware specification for the experiments was a Dell R730 system, which used Xeon E5-2660, 2.6 GHz, 64 GB memory, and 800 GB SSD. For performing deep learning, the experiments were conducted with a CPU only, excluding the GPU (graphics processing unit). When measuring the analysis performance, a 10-fold cross-validation protocol was used. In general, when the analysis performance of the system is measured, because the accuracy used has an accuracy paradox issue [36,37], it is better not to use it for measuring classification performance. Therefore, in this paper, the main metric is the F1 score, and the precision and recall were measured as supplementing metrics. The definitions of these metrics are as follows:

(5) $$Precision=\frac{tp}{tp+fp},$$

(6) $$Recall=\frac{tp}{tp+fn},$$

(7) $$F1\;score=\frac{2}{\frac{1}{recall}+\frac{1}{precision}}=\frac{2\;\cdot precision\cdot recall}{precision+recall},$$

That is, precision is the proportion of instances that is truly of a class divided by the total instances classified as that class, and the recall is the proportion of instances classified as a given class divided by the actual total in that class. The F1 score is a harmonic average of precision and recall, where its best value is 1 and worst is 0. *tp*, *fp*, and *fn* stand for true positive, false positive, and false negative, respectively.

### 3.3. Experiment 1: Searching for the Optimal Degree of Quantization

In Experiment 1, to find an optimal encoding base number, *baseN*, for the Wave2vec model, multiple versions of the model using various encoding base numbers were constructed, and the changes in performance for classifying EEG were observed. In general, the resolution or sampling rate in the data communication or signal processing refers to how finely the time is divided to represent the data by having the time axis as a variable. However, the resolution in this experiment represents the y-axis of the signal, i.e., degree of quantization of signal amplitude. The resolution in this experiment is defined by the encoding base number, which represents the size of the encoding target symbol set. The bottom left panel of Figure 2 shows an example of quantization by using 16 symbols from 0 to F for the amplitude of the signal via encoding with the base number F. In general, as the base number of quantization is increased, the data loss is decreased, but the effects of encoding, such as reduction of complexity and improvement of analysis speed, decreased. Inversely, if the base number is decreased, the analysis time is reduced due to the decrease of complexity, but because data loss becomes severe, the analysis performance will drop. Therefore, a base number of appropriate tradeoff must be determined.

The time interval of the X-axis is a variable, and thus, a different interval can be chosen depending on the data type and encoding method. There will be a different optimal encoding variable, depending on whether the type of signal data processed is EEG, heart rate, stock price data, climate data, or amount of precipitation.

For this experiment, targeting the UCI alcohol EEG data, 13 different Wave2vec EEG classifiers were constructed by changing the encoding base number from 8 to 1024, i.e., *baseN* = {8, 16, 32, 48, 64, 96, 128, 192, 256, 384, 512, 768, 1024}. For each EEG classifier, by learning two EEG class training data, i.e., alcohol addiction patient class and normal control class, the alcohol addiction EEG model and normal EEG model were created, respectively, thereby creating 26 EEG models in total. In the learning stage, CBOW was used for the deep learning architecture for the learning of encoded sequence of symbols, the sliding window size was set at 10, the dimension representing the length of targeting vector was set at 50, and the minimum frequency for filtering was set at 1. The experiment was carried out with a method of classifying 10,000 testing samples as normal EEG or alcohol addiction EEG every time by using the 10-fold cross-validation protocol. In this study, F1 score was measured as the major metric and the precision and recall were measured as supplementary metrics.

**Figure 10:**
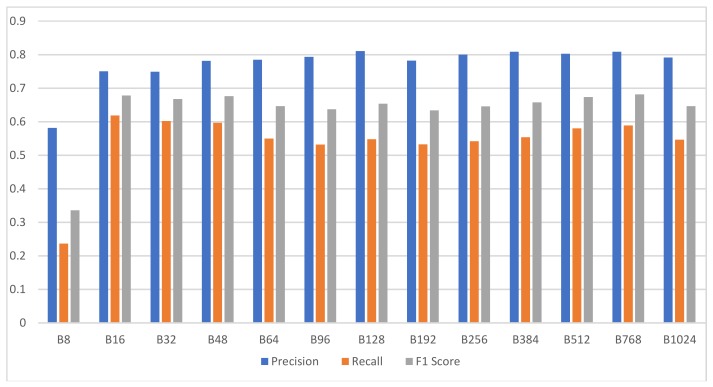
Classification performance of Wave2vec by encoding resolution. X-axis represents the encoding base numbers and y-axis represents the score, whose scale is from 0 to 1.

The bar chart of Figure 10 summarizes the experimental results for the analysis performance comparison of 13 difference versions of Wave2vec classifiers, in which the encoding base number was changed, and displays the precision, recall, and F1 score of each system. From these results, it can be observed that the proposed encoding-based Wave2vec time series classifier shows inferior performance when using sparse encoding, but as the number of encoded words increases, the performance is improved quickly; the precision becomes 78.47% for *baseN* = 64 encoding and 81.05% for *baseN* = 128 encoding, showing a high performance for *baseN* of 64 or above. F1 score, which is the harmonic mean of precision and recall, is shown as gray bars in this chart, and it continuously shows good performances for *baseN* = 16 or higher, thereby showing that higher *baseN* encodings are not very meaningful. As a side note, the best accuracy was measured as 68.55% when *baseN* = 512.

Although there were some differences in the type and number of channels used, in previous research by [38] analyzing the same data, the accuracy and precision were 0.650 ± 0.055 and 0.65 ± 0.04, respectively, in *k*-nearest neighbors (*k*NN) when *k* = 1; 0.662 ± 0.053 and 0.65 ± 0.04, respectively, in *k*NN when *k* = 10; and 0.78 ± 0.112 and 0.81 ± 0.10, respectively, when Naïve Hubness Bayesian *k*NN was used. Furthermore, as for the classification accuracy of channel CP6 in the study done by Zhu [33], accuracy and probably precision were 62.7% and 67.8% for *k*NN and support vector machine (SVM), respectively, when the sample entropy method was used, and 77.2% and 79.1% for *k*NN and SVM, respectively, when the horizontal visibility graph entropy (HVGE) approach was used. Considering these, it was found that the analysis performance of the proposed encoding-based Wave2vec time series classifier was similar to or slightly inferior to those in previous research due to the data loss in the encoding stage. These results were somewhat anticipated, as they were inevitable results of encoding in order to achieve the main goals of this study: to improve the analysis speed by reducing the complexity through encoding; to increase the readability of the analysis process and results; and to increase the capabilities for recognition and identification of effective patterns. Nevertheless, an additional study will be necessary to minimize or eliminate the performance deterioration due to these factors.

### 3.4. Experiment 2: Comparison with Other Deep Learning Classifiers

In Experiment 2, comparisons were conducted for the analysis performance, the memory size required for analysis, and the analysis time between the EEG analysis systems using the Wave2vec model constructed by applying the encoding parameters found in Experiment 1 and the conventional deep learning classifiers implemented by using the three major deep learning architectures—CNN, RNN, and DNN.

**Figure 11:**
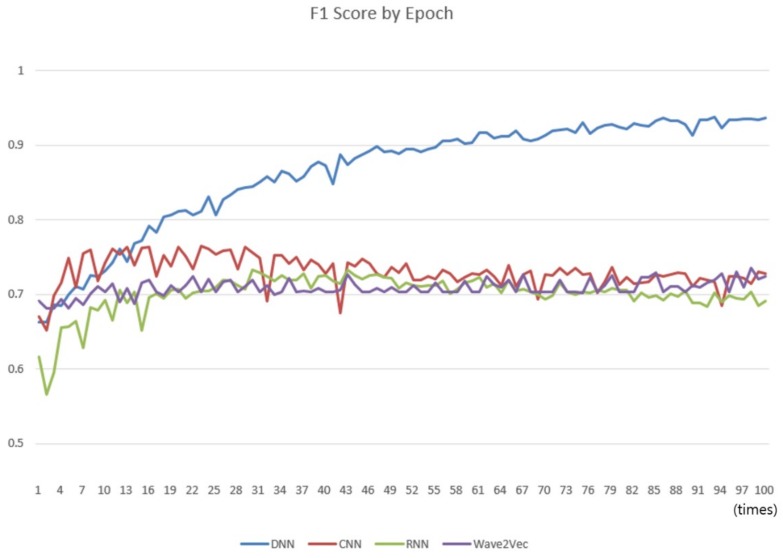
Comparison of the change of F1 scores of the four classifiers as the number of epochs varies from 1 to 100. The X-axis represents the number of epochs and y-axis represents score, whose scale is from 0 to 1.

The comparisons were carried out with the classifiers of conventional CNN, RNN, and DNN models to compare the classification performances of the Wave2vec time series classifier of encoding *baseN* = 64 constructed in Experiment 1. A cycle of learning the entire training data once is called an epoch, and while increasing the number of epochs from 1 to 100, the performance of each classifier was measured every time, and a chart was produced as shown in Figure 11. In every run, 0.2 was applied for the dropout. In the chart, the performance of Wave2vec is better than those of the other models for small epochs of 1–3, which is an early stage of repetition of learning, but the performance of CNN is better when it is 4–10, and that of DNN is better thereafter up to 100. Because the Wave2vec became similar to the other models (CNN, RNN, and DNN), excluding DNN after the epoch of 10, it was found that there was not much significant difference in the performances. In the case of DNN, as the epoch becomes close to 100, its performance is continuously increased to 90 or higher. Unlike other deep learning models, however, in DNN, which is more like the traditional neural network rather than the deep learning technique, the ‘*early stop*’ should be considered and high performances by high iteration number should be considered as the result of an overfitted model.

An interpretation of this result is that, because the CBOW is originally designed for constructing vector models rather than for classification or regression calculation, even the accuracy of constructed vector models improves according to the increase of the number of epochs; the speed of enhancing is too slow to influence the performance of classification directly. However, considering that a lot of time and computing cost are required for conducting learning for one epoch (refer to Table 2 for training time), Wave2vec showed better performances at initial stages of learning, i.e., at low epoch stages from one to three. Compared to the cases of the other systems, it is an advantage that a system that reached a certain level of performance with high speed can be constructed. Such a characteristic is appropriate for large-capacity analysis or real-time analysis systems.

Analysis performance is important, but so are the training (learning) speed and the speed of performing the diagnosis, i.e., classification, with respect to the new input data. Therefore, another experiment was carried out to compare the training speed and diagnosis speed with those of the other systems. The proposed Wave2vec model not only reduces the size of signal data noticeably through data encoding, but also reduces the calculation complexity. Thus, the turnaround time is also reduced when performing learning and analysis.

To measure the training speed and diagnosis speed, the changes of data size that affected the loading time were measured. The raw data of the CP6 channel for alcohol addiction EEG used in the measurement consisted of instances of 11,060 text files. Each instance included 250 EEG amplitude values. Among the UCI data, the size of the CP6 channel used in the experiment was 44,672,000 bytes on the file system, and when loaded on the system, if a value of each amplitude, i.e., real number, is loaded as a short float number, which is of the short double type, 4 bytes is required for one real number, and thus, total memory of 11,325,440 bytes will be used just for loading. However, when the same data are loaded after encoding, only 2,831,360 bytes are used, because one symbol can be loaded by only 1 byte. Table 2 shows the training time for the entire training data of the Wave2vec and three deep learning classifiers, and the testing time for returning a classification result when a new testing data is input. The encoding of Wave2vec was done with *baseN* = 6, and measurement was taken for only one epoch. Only the CPU was used, and the GPU was excluded. The execution was carried out by using the *Keras* [34] on a Dell R730 system with Xeon E5-2660, 2.6 GHz, 64 GB memory, and 800 GB SSD.

The training time includes the time for loading, time for encoding, and time for performing the fitting of the neural net. The encoding time was added to the Wave2vec because encoding is performed only for the Wave2vec model, but it can be observed that the time required for encoding is very short compared with the time required for the other tasks. As shown in this table, owing to simplification of data through encoding, the training time is noticeably decreased compared with the cases of the other models. Particularly, compared to the RNN method, which is the conventional deep learning system showing the best accuracy for sequence classification, there is a vast difference in the training time. In the measurement of testing time, the Wave2vec shows speed improvement. In the case of Wave2vec, as classification is carried out with vector calculations for as many times as the number of classes, twice in the case of this experiment, without involvement of the neural net during testing, we observed that the response is short compared to those of the other models in which the neural nets are involved. Considering that one instance of testing data used in this experiment is an analysis time for EEG data of 1 s duration for one channel, it can be expected that the analysis time difference between the models will be larger in an actual analysis environment because, normally, 32 channels or 64 channels are used in a real EEG analysis system, and EEG acquired for 6–8 h is sometimes analyzed in the case of sleep EEG analysis.

The fast processing speeds of training and testing are essential functions in the development of fast time series data analysis systems, such as the real-time EEG analysis system for fast brain disease prediction and a large-capacity EEG data analysis system for sleep EEG research.

### 3.5. Experiment 3: Recognition and Identification of Effective Patterns through Model Visualization

In Experiment 3, we showed how we recognize and identify the most effective wavelet patterns for prediction and diagnosis from the training data. We built a Wave2vec time series classifier using the optimal encoding parameters found during Experiment 1 and created two EEG models representing the two EEG classes. The alcoholic EEG model is generated by learning alcoholic EEG data, and the normal control EEG model is made by learning normal control EEG training data.

**Figure 12:**
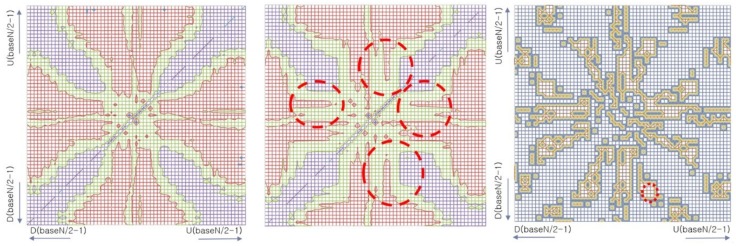
Visualized normal EEG model (**left**) and alcoholic EEG model (**center**). Different parts of the two models are marked by red circles (**center**). Effective parts for classifying the two models were accentuated by performing XOR the two models (**right**).

The pattern recognition is achieved by visualizing two class models: the alcohol EEG model and normal EEG model. These EEG models are in the form of a wave vector tables that consist of wave vectors of all encoded wavelet patterns. Therefore, visualizing the two wave vector tables from the two models can provide an overview of the models and insight of the difference between the two classes. We employed the “heat map” visualization technique [39] (see the Figure 12 below), because the vector table is two-dimensional data, the relations between patterns are easy to understand, and the difference in structures between the models are easy to recognize.

Comparison and classification of patterns in a wave vector table are possible once the patterns are in vector format. However, direct comparison or operations among patterns from multiple wave vector tables are not possible because they exist in different vector spaces. Instead, comparison between vector cluster sets across wave vector tables are valid and applicable because no direct vector comparison or operations across wave vector tables are needed. In this experiment, therefore, instead of visualizing raw vector tables of the two models, we visualized them as similarity matrices to represent the probability of clustering between two wavelet patterns with a heat map. The similarity matrix is the complete result of clustering every two wavelet pattern combinations in order to visualize the difference of the two models. Thus, the matrix is diagonally symmetric. From the heat maps of the two models, many analyses and interpretations are possible, such as “the probability of appearance of a pattern ‘pattern A is followed by pattern B’ is higher in model one while lower in model two”, or “more chance of serial two patterns from high amplitude change range in model 1 than 2”, and so on. Furthermore, because the wave vector indexes of interesting patterns are retrievable from the heat map, it is possible to identify what the actual patterns are by referring to the pattern dictionary.

The left and center heat maps of Figure 12 are visualized similarity matrices of the two EEG class models: the left is from the normal control model and center is from alcoholic model. Both models are encoded by *baseN* = 64. The horizontal and vertical axes represent patterns where the bottom left locates greater dropping patterns, which were symbolized by *D*(*baseN-a*), at the top right are greater rising patterns that were symbolized by *U*(*baseN-a*), and the central area is where slight changing patterns, such as *U*(0) and *D*(0), are mapped. Each pixel of the heat map represents the similarity, which is the probability of clustering, between the two associated patterns. The color shows the degree of similarity, where red means higher and blue means lower similarity. The practical meaning of similarity between patterns in a vector space means the probability of co-occurrence and substitution potential of the two patterns, calculated by cosine vector similarity, expressed by Equation (4).

This visualization presents an overview of the two models from each EEG class and provides insight of the difference of structures between the two EEG class models. An example of different areas where most effective patterns are roughly marked by a dashed red circle is in the similarity matrix in the center image of Figure 12. These areas can be systematically emphasized by performing the XOR operation, which will highlight only the differences between the two similarity matrices. The right image of Figure 12 is the result of the XOR operation of the two similarity matrices shown by left and center images. From this result, the index of patterns in the wave vector table can be easily retrieved, from which the actual patterns can be identified by searching the pattern symbol dictionary. For instance, from the marked area in the right similarity matrix, we can say that the probability of occurrence of slow-rising amplitude patterns, from *U*7 to *U*10, followed by rapid dropping amplitude patterns, from *D*21 to *D*24, is different in the two classes. The likelihood of seeing these patterns is higher in the normal control class EEG than in the alcoholic class EEG.

In this example, we described how two adjacent pattern sequences that are effective in classifying the classes are found from the proposed encoding-based Wave2vec time series classifier by using visualization, with two two-dimensional *baseN* × *baseN* similarity matrices of each model. While this experiment shows only the case of two-dimensional clustering, this approach can also be applicable for searching for longer effective pattern sequences by employing higher-dimensional clustering methods between patterns.

As demonstrated in this experiment, the proposed Wave2vec model has a strong capability in presenting and explaining the training process and reason for classification, while conventional deep learning-based time series classifiers do not. This capability is more valuable in biomedical applications, where readability, understandability, and facility of explanation are important for prediction, diagnosis, and decision-making.

## 4. Discussion

The current approach has several issues and limitations.

1. The current proposed models did not employ any disease-specific context knowledge for enhancing analysis performance. The model is a general-purpose time series classifier, and its main goals are solving specific issues in analyzing bio-signal with conventional deep learning models, such as removing black boxes, reducing complexity, and recognizing and identifying important patterns. For real application to prediction or diagnosis of brain disease, such as dementia or alcoholism, more complicated knowledge and logic of the targeted disease, such as the relationship between the disease and the geometric information of EEG sensing spots, connections between sensing spots on the scalp, and democratic knowledge of the testing person, should be melted at the tokenizing phase.
2. The analysis performance of the model, in terms of accuracy, is similar to those of conventional deep learning models and inferior to that of the deep neural network (DNN) if overfitting is ignored.
3. Overfitting should be removed. As the number of epochs increases, the DNN tends to converge to a perfect status, which is overfitting. The process for data augmentation and the method of regularization, such as parameter tweaking, need to be studied.
4. Unlike conventional deep learning models, the performance of the current approach is not affected directly, or affected inefficiently, by increasing the number of iterations of whole data training. This is caused by replacing large portions of the deep learning process for feature selection and classification with vector operations to reduce the number of black boxes and improve readability.
5. A hybrid model, which includes both conventional deep learning modules and the proposed Wave2vec modules, should be considered because the most important values of classifiers, such as accuracy, transparency, readability, visibility, and high speed, will vary depending on the application areas.

## 5. Conclusions

In this study, a novel deep learning model was proposed to solve some problems that occur when analyzing bio-signal data, such as EEG and ECG, for prediction and diagnosis of diseases with the conventional deep learning techniques. Such problems include: (1) difficulty explaining and understanding the analysis process and results due to the black box feature extraction process and learning; (2) difficulty recognizing and identifying effective patterns; and (3) high complexity of calculations due to real number time series data processing. The proposed model is an encoding-based Wave2vec time series classifier, and its basic concept is that it produces symbols for the wavelet patterns that compose the bio-signals, and by learning the emerging contexts and relationships of respective patterns, it represents the patterns and signals as multi-dimensional vectors. To this end, an encoding technique, which is a data communication and compression technique, was applied, and so were deep learning techniques for natural language processing, such as CBOW.

In this paper, we showed that the above three issues of deep learning models were solved by the proposed model through three experiments. The readability was improved for the feature extraction process and result, which were black boxes in the conventional deep learning models, and this was confirmed by letting the user select the target feature of encoding by him/herself and constructing a wave vector table, which is a learning result of simultaneous emerging information of patterns through CBOW. Furthermore, it was possible to recognize the effective patterns through vectorization of all emerging patterns and visualization of wave vector tables, and to identify the effective patterns through reverse-tracking using the wave vector table index. Moreover, through actual learning and diagnosis time measurement experiments, it was confirmed that the time required for learning, prediction, and diagnosis was reduced drastically by improving the calculation complexity through simplification of data. Such characteristics of the proposed model are the functions needed in the medical information system, wherein the reason and explanation are required for the analysis of data and diagnosis of diseases, and actual effective data must be extracted and provided.

In addition, fast analysis speed is an essential function in the development of real-time prediction and diagnosis systems, such as a simple EEG analysis system and large-capacity sleep EEG analysis system. In addition, in the experiment for examining the changes of analysis performance according to encoding, it was shown that there was almost no difference in the analysis performance compared to the conventional deep learning systems, even if quantization of bio-signals is conducted with about 32 symbols only. In the experiment comparing the analysis performance with that of conventional deep learning systems using F1 measures, a better performance was observed when the epoch was low, and a similar performance was observed at high epochs.

The proposed encoding-based Wave2vec time series classifier is a general-purpose algorithm that can be applied to analysis of all wave type time series data, such as financial information, sound data, and climate data, as well as bio-signal analysis. Nevertheless, because it was not customized for certain areas, like alcohol addiction, it was confirmed that its analysis performance result was somewhat lower than those of conventional studies. Therefore, for future research, a follow-up study will be carried out for a method of optimizing this system by applying the context knowledge according to the application area. The study must be carried out for optimization by analyzing the characteristics of various types of time series data, such as EEG, ECG, financial data, and climate data, and by finding optimal encoding features and parameters, and an optimal deep learning model and learning method suitable for respective data.

## Figures and Tables

**Table 1:** Architectures of three deep learning EEG classifiers implemented for the experiment.

CNN-Based EEG Classifier	RNN (LSTM)-Based EEG Classifier	DNN-Based EEG Classifier
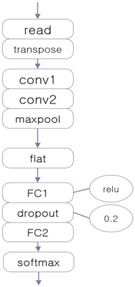	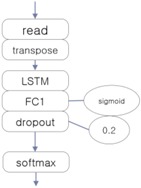	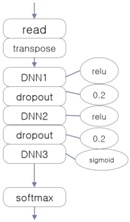

**Table 2:** Comparison of training time and testing time.

	Training Time (Seconds per Epoch)			Testing Time (Seconds per Instance)		
	Loading	Encoding	Learning	Loading	Encoding	Classifying
Wave2vec	5.650	2.250	23.020	0.005	0.000	0.059
CNN	5.650	-	194.030	0.005	-	0.390
RNN	5.650	-	323.130	0.005	-	0.574
DNN	5.650	-	206.394	0.005	-	0.480

## References

[1] Wilson R., Willis J., Gearry R., Skidmore P., Fleming E., Frampton C., Carr A. (2017). Inadequate vitamin C status in prediabetes and type 2 diabetes mellitus: Associations with glycaemic control, obesity, and smoking. Nutrients.

[2] Kim H., Chun H.-W., Kim S., Coh B.-Y., Kwon O.-J., Moon Y.-H. (2017). Longitudinal Study-Based Dementia Prediction for Public Health. Int. J. Environ. Res. Public Health.

[3] Strichartz R.S. (2003). A Guide to Distribution Theory and Fourier Transforms.

[4] Cohen M.X. (2014). Analyzing Neural Time Series Data: Theory and Practice.

[5] Schomer D.L., Da Silva F.L. (2012). Niedermeyer’s Electroencephalography: Basic Principles, Clinical Applications, and Related Fields.

[6] An X., Kuang D., Guo X., Zhao Y., He L. (2014). A deep learning method for classification of EEG data based on motor imagery. Proceedings of the International Conference on Intelligent Computing.

[7] Tabar Y.R., Halici U. (2016). A novel deep learning approach for classification of EEG motor imagery signals. J. Neural Eng..

[8] Ren Y., Wu Y. Convolutional Deep Belief Networks for Feature Extraction of EEG Signal. Proceedings of the Neural Networks (IJCNN).

[9] Hussein R., Palangi H., Ward R., Wang Z.J. (2018). Epileptic Seizure Detection: A Deep Learning Approach. arXiv.

[10] Marcus G. (2018). Deep Learning: A Critical Appraisal. arXiv.

[11] Kivipelto M., Ngandu T., Laatikainen T., Winblad B., Soininen H., Tuomilehto J. (2006). Risk score for the prediction of dementia risk in 20 years among middle aged people: A longitudinal, population-based study. Lancet Neurol..

[12] Colon I., Cutter H.S., Jones W.C. (1982). Prediction of alcoholism from alcohol availability, alcohol consumption and demographic data. J. Stud. Alcohol..

[13] Xing Z., Pei J., Keogh E. (2010). A brief survey on sequence classification. ACM Sigkdd Explor. Newsl..

[14] Lesh N., Zaki M.J., Ogihara M. Mining features for sequence classification. Proceedings of the Fifth ACM SIGKDD International Conference on Knowledge discovery and Data Mining.

[15] Zhang Y., Yang S., Liu Y., Han B., Zhou F. (2018). Integration of 24 Feature Types to Accurately Detect and Predict Seizures Using Scalp EEG Signals. Sensors.

[16] Yuan Y., Xun G., Suo Q., Jia K., Zhang A. Wave2vec: Learning deep representations for biosignals. Proceedings of the 2017 IEEE International Conference on Data Mining (ICDM).

[17] Mikolov T., Chen K., Corrado G., Dean J. (2013). Efficient estimation of word representations in vector space. arXiv.

[18] Sun R., Alexandre F. (2013). Connectionist-Symbolic Integration: From Unified to Hybrid Approaches.

[19] Hall L.O., Romaniuk S.G. A Hybrid Connectionist, Symbolic Learning System. Proceedings of the AAAI.

[20] Moreno P.J., Stern R.M. Sources of degradation of speech recognition in the telephone network. Proceedings of the 1994 IEEE International Conference on Acoustics, Speech, and Signal Processing (ICASSP-94).

[21] Wright J., Yang A.Y., Ganesh A., Sastry S.S., Ma Y. (2009). Robust face recognition via sparse representation. IEEE Trans. Pattern Anal. Mach. Intell..

[22] Liu C., Wechsler H. (2002). Gabor feature based classification using the enhanced fisher linear discriminant model for face recognition. IEEE. Trans. Image Process..

[23] Rabiner L.R., Gold B. (1975). Theory and Application of Digital Signal Processing.

[24] Mogul J.C., Douglis F., Feldmann A., Krishnamurthy B. (1997). Potential benefits of delta encoding and data compression for HTTP. ACM SIGCOMM Comput. Commun. Rev..

[25] Mladenic D., Grobelnik M. Word sequences as features in text-learning. Proceedings of the 17th Electrotechnical and Computer Science Conference.

[26] Sharma A., Dey S. An artificial neural network based approach for sentiment analysis of opinionated text. Proceedings of the 2012 ACM Research in Applied Computation Symposium.

[27] Zhou C., Cule B., Goethals B. (2016). Pattern based sequence classification. IEEE Trans. Knowl. Data Eng..

[28] Kim S., Yeo W., Lee J., Kim K.-H. Linguistic Feature Learning for Technological Information Detection. Proceedings of the International Conference on Convergence Content (ICCC2012).

[29] Salton G., McGill M. (1983). Introduction to Modern Information.

[30] Kuang S., Davison B.D. (2017). Learning Word Embeddings with Chi-Square Weights for Healthcare Tweet Classification. Appl. Sci..

[31] Begleiter H. EEG Database Data Set. https://archive.ics.uci.edu/ml/datasets/EEG+Database.

[32] Zhang X.L., Begleiter H., Porjesz B., Litke A. (1997). Electrophysiological evidence of memory impairment in alcoholic patients. Biol. Psychiatry.

[33] Zhu G., Li Y., Wen P.P., Wang S. (2014). Analysis of alcoholic EEG signals based on horizontal visibility graph entropy. Brain Inform..

[34] Chollet F. (2015). Keras: Deep Learning Library for Theano and Tensorflow.

[35] DeepLearning4j Deep Learning for Java. https://deeplearning4j.org/.

[36] Wikipedia Accuracy Paradox. https://en.wikipedia.org/wiki/Accuracy_paradox.

[37] Akosa J. Predictive Accuracy: A Misleading Performance Measure for Highly Imbalanced Data. Proceedings of the SAS Global Forum.

[38] Buza K.A., Koller J. (2016). Classification of electroencephalograph data: A hubness-aware approach. Acta Polytech. Hung..

[39] Wilkinson L., Friendly M. (2009). The history of the cluster heat map. Am. Stat..

